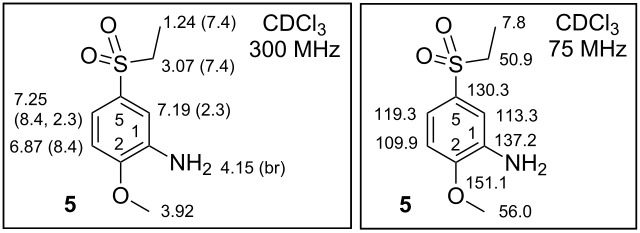# Synthesis of 5-(ethylsulfonyl)-2-methoxyaniline: An important pharmacological fragment of VEGFR2 and other inhibitors

**DOI:** 10.3762/bjoc.9.20

**Published:** 2013-01-25

**Authors:** Miroslav Murár, Gabriela Addová, Andrej Boháč

**Affiliations:** 1 Department of Organic Chemistry, Faculty of Natural Sciences, Comenius University, Mlynská dolina, 842 15 Bratislava, Slovakia; 2 Institute of Chemistry, Faculty of Natural Sciences, Comenius University, Mlynská dolina, 842 15 Bratislava, Slovakia; 3 Biomagi Ltd. Mamateyova 26, 851 04 Bratislava, Slovakia

**Keywords:** angiogenesis, pharmacophoric ligand, synthesis of 5-(ethylsulfonyl)-2-methoxyaniline, VEGFR2 tyrosine kinase inhibitors

## Abstract

**Background:** 5-(Ethylsulfonyl)-2-methoxyaniline (**5**) is part of the structure in 131 compounds possessing different biological activities. In most cases, they have antitumor properties (112 compounds). Other compounds are described as cardiovascular agents, ion-channel blockers, nervous-system blockers, anti-inflammatory agents, or antidiabetic, antiosteoporotic and hypolipemic species. Compound **5** is a precursor of different protein-kinase inhibitors or enzyme modulators (EGFR, PDGFR, ckit, CDK 2 and 4, MMPs 2, 3, 9 and 13, etc.). The structure of **5** represents a fragment for several powerful inhibitors of VEGFR2, a key angiogenic receptor. Antiangiogenic inhibitors slow down or stop new blood-vessel formation from pre-existing vasculature. Some antiangiogenic drugs inhibiting the VEGFR2 receptor are successfully used in clinics for the treatment of several types of tumours in synergy with chemotherapy (e.g., Nexavar^®^ from Bayer, Sutent^®^ from Pfizer and Votrient^®^ from GlaxoSmithKline, approved by the FDA in 2005, 2006 and 2009, respectively). The structure of **5** is an important pharmacophoric fragment of potent VEGFR2 inhibitors (e.g., **AAZ** from PDB complex 1Y6A, enzymatic IC_50_ = 22 nM). Up to now, 25 VEGFR2 inhibitors possessing a fragment of **5** can be found in the literature. Despite the high significance of 5-(ethylsulfonyl)-2-methoxyaniline (**5**) its preparation has not yet been described.

**Results:** Here we have developed a convenient synthesis of important polyheterosubstituted aniline **5** starting from commercially available 4-methoxybenzene-1-sulfonyl chloride (**1**) in four steps and 59% overall yield. The target 5-(ethylsulfonyl)-2-methoxyaniline (**5**) and its synthetic intermediates **2**–**4** together with a new compound 5-(ethylsulfonyl)-2-methoxy-1,3-dinitrobenzene (**4a**) have been precisely physicochemically characterised.

## Introduction

5-(Ethylsulfonyl)-2-methoxyaniline (**5**) is a starting material and a structural fragment of 131 compounds possessing different biological activity, mostly described as antitumor agents (112 compounds) as well as cardiovascular agents, ion-channel blockers, nervous-system blockers, anti-inflammatory agents, and antidiabetic, antiosteoporotic or hypolipemic species. Compound **5** is used for the development of small organic compounds, i.e., modulators targeting a broad spectrum of important human protein receptors or enzymes, e.g., VEGFR2, EGFR, PDGFR, TEK kinase, ckit, EphB4, ErbB-2 receptor tyrosine kinase, cyclin-dependent kinases 2 and 4, neu receptor, polo-like kinase 1, alpha and beta adrenoreceptors, glycogen phosphorylase, IMP dehydrogenase, MMPs 2, 3, 9 and 13, etc. [1].

Vascular endothelial growth factor (VEGF-A) is a homodimeric glycoprotein and thought to be the key signalling molecule of angiogenesis, i.e., the formation of new blood vessels from pre-existing ones. Angiogenesis is essential for cancers to develop from a small size. VEGF-A binds to two VEGF receptors that are expressed on the surface of vascular endothelial cells (VEGF receptor-1 and VEGF receptor-2). New vasculature is formed in and around the tumour, allowing it to grow exponentially and form life-threatening metastasis [2–3].

The development of 2-anilino-5-aryloxazole containing inhibitors of VEGFR2 receptor led to potent antiangiogenics at both the enzymatic and cellular levels. Twenty-two derivatives of *N*-(5-(ethylsulfonyl)-2-methoxyphenyl)-5-phenyloxazol-2-amines with determined enzymatic (IC_50_, VEGFR2) and cellular activities (IC_50_, hu-HUVEC/VEGF) were described. Among them *N*-(5-(ethylsulfonyl)-2-methoxyphenyl)-5-(3-(pyridin-2-yl)phenyl)oxazol-2-amine (**AAZ**) proved to be the most promising compound (IC_50_: 22 nM, VEGFR2) [4]. The binding mode of the **AAZ** ligand in the VEGFR2 receptor has been solved by X-ray crystallography and is available in the Protein Data Bank (PDB: 1Y6A). The complex 1Y6A contains the intracellular tyrosine kinase domain of the VEGFR2 receptor, which accommodates two conformers of the **AAZ** ligand [5].

**Figure 1:**
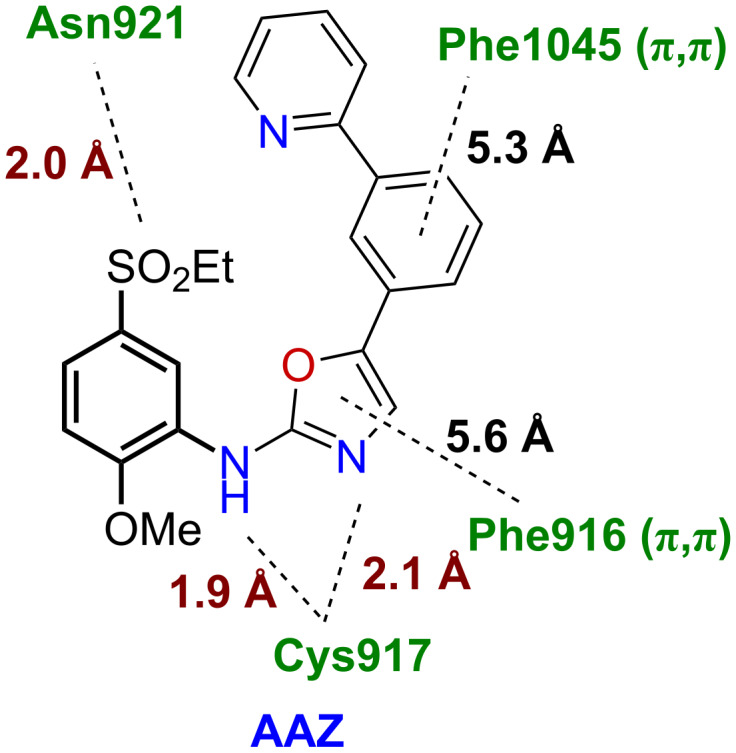
The **AAZ** ligand conformer from PDB complex 1Y6A and its VEGFR2 intermolecular interaction map depicting three hydrogen bonds and two stacked (π,π) interactions. No lipophilic interactions are shown here. The part of the skeleton **AAZ** in bold represents the fragment originating from the precursor **5.** The interaction analysis has been performed by software Discovery Studio Visualizer 3.1 [6].

Compound **5** is an important precursor for the synthesis of the **AAZ** inhibitor and its derivatives. The fragment of **5** in the **AAZ** skeleton has pharmacophoric properties and ensures some critical intermolecular interactions with the amino acid residues of the VEGFR2 protein (e.g., PDB: 1Y6A; Asp921 and Cys917), (Figure 1).

Based on the above knowledge, we have recently developed three VEGFR2 inhibitors, i.e., derivatives of **AAZ** that contain a structural fragment of **5** (IC_50_ = 12.8; 14.7 and 87.3 nM) [7]. Several years ago Sigma-Aldrich offered 2-amino-4-(ethylsulfonyl)phenol, which we transformed to the required 5-(ethylsulfonyl)-2-methoxyaniline (**5**) in three simple synthetic steps (N-acetylation, O-methylation, N-acetyl deprotection). Later on 2-amino-4-(ethylsulfonyl)phenol was discontinued from current commercial sources. Compound **5** is still available in small 1 g quantities but at a price that was unfavourable (e.g., as part of the Aldrich^CPR^ collection) [8]. Nevertheless, compound **5** is cited in the database Reaxys (Reaxys Registry Number 783509, 37 reactions) and SciFinder Scholar database (CAS [5339-62-8], 191 reactions). In all cases, this compound was mentioned exclusively as a starting material or reactant [9]. Surprisingly the synthesis of this highly important 3-heterosubstituted aniline **5** has not yet been published in research journals or patents, and the properties of compound **5** have not been completely described. We could find only a few of the physicochemical characteristics for **5**. Experimental IR and ^1^H NMR (CDCl_3_) spectra are mentioned only in the SciFinder database and originated from Bio-Rad Laboratories data collection [9].

Considering the above situation, we decided to develop a smart synthesis of substituted aniline **5** from available starting materials and determine all of the important physicochemical characteristics of the compounds from the successful synthetic pathway.

## Results and Discussion

**Scheme 1:**
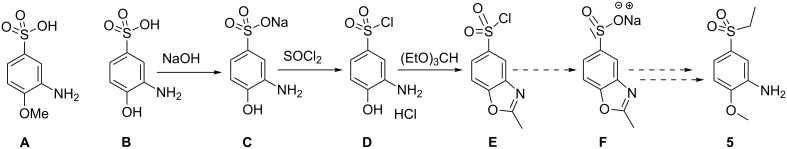
The structures of commercially available arylsulfonic acids **A** and **B** both appropriately substituted for the synthesis of product **5**. An unsuccessful synthesis of compound **5** starting from commercially more suitable 3-amino-4-hydroxybenzenesulfonic acid (**B**).

In order to find a convenient methodology for the synthesis of the desired compound **5**, we decided to use an appropriately substituted commercially available starting material. Sigma-Aldrich offers two arylsulfonic acids possessing all three hetero-substituents at the correct positions within a benzene ring. 3-Amino-4-methoxybenzenesulfonic acid (**A**) and its demethylated precursor 3-amino-4-hydroxybenzenesulfonic acid (**B**) were selected. In particular, compound **B** is available in large quantities (250 g) at a very reasonable cost. Several attempts were carried out to transform the sulfonyl group of 3-amino-4-hydroxybenzenesulfonic acid (**B**) to the required ethylsulfonyl functionality. Sodium 3-amino-4-hydroxybenzenesulfonate (**C**) was quantitatively obtained from 3-amino-4-hydroxybenzenesulfonic acid (**B**) by treatment with NaOH. The sodium salt **C** was subsequently converted to the hydrochloride salt of 3-amino-4-hydroxybenzene-1-sulfonyl chloride (**D**) by treatment with thionyl chloride at room temperature. The amino group of **D** was protected in the form of a 2-methylbenzo[*d*]oxazole-5-sulfonyl chloride (**E**) by reaction of **D** with (EtO)_3_CH in the presence of *p*-TsOH. Reduction of sulfonyl chloride **E** to sodium sulfinate **F** by Na_2_SO_3_ and Na_2_CO_3_ and its reaction with ethyl iodide proceeded as nonselective reactions, which excluded this methodology from further efforts (Scheme 1).

**Scheme 2:**
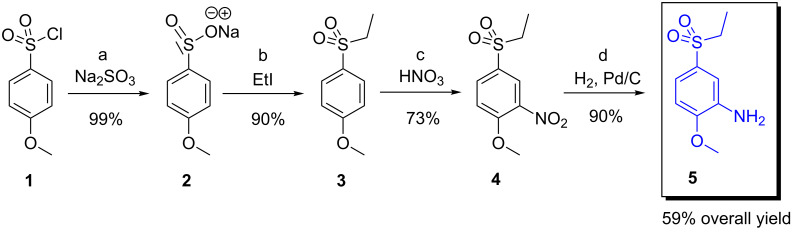
Synthetic pathway starting from the commercially available sulfonyl chloride **1** leading to the required 5-(ethylsulfonyl)-2-methoxyaniline (**5**). Reaction conditions: (a) Na_2_SO_3_ (2 equiv), NaHCO_3_ (2 equiv), H_2_O/THF (10:1), 0 °C/rt overnight, yielded 99% of **2**; (b) MeOH, EtI (2.5 equiv), reflux 2 h, 90% of **3**; (c) conc. HNO_3_, 100 °C, 2 h, 73% of **4** (under different conditions: conc. HNO_3_/H_2_SO_4_ (1:1, v/v), 60 °C, 1 h, yielded 54% of dinitro derivative **4a**); (d) 10% Pd/C, H_2_, 2 days, 90% of **5**. Compound **5** was prepared in 58.5% overall yield.

These findings suggested that the free amine in **B** was considered to be a weak point of this first synthetic route. Therefore, we explored a second pathway starting from commercially available 4-methoxybenzene-1-sulfonyl chloride (**1**). Compound **1** can be prepared easily from methoxybenzene (anisole) and ClSO_3_H in 88% yield [10]. Following the second proposed methodology, the starting material **1** provided 5-(ethylsulfonyl)-2-methoxyaniline (**5**) in four synthetic steps and 59% overall yield. No chromatography was needed in order to obtain the synthetically pure product (Scheme 2).

## Conclusion

Even though 5-(ethylsulfonyl)-2-methoxyaniline (**5**) is a very important compound frequently used as a starting material, reagent or pharmacophoric fragment, its synthesis has not yet been described. Because the aniline derivative **5** possesses three heterosubstituents on the benzene ring, a convenient starting material had to be selected for its preparation. We have developed a novel and the only known preparation of **5**, in 59% overall yield in four steps starting from commercially available 4-methoxybenzene-1-sulfonyl chloride (**1**). The starting material **1** can be obtained from well-known methoxybenzene (anisole) by a one-step reaction of anisole with sulfuryl chloride in 88% yield [10]. The synthesis of 5-(ethylsulfonyl)-2-methoxyaniline (**5**) can be utilized for the development of biologically active compounds as well as for different synthetic purposes.

## Experimental

^1^H and ^13^C NMR spectra were recorded on Varian Gemini (300 MHz and 75 MHz, respectively), chemical shifts are given in parts per million (ppm), and tetramethylsilane was used as an internal standard and DMSO-*d_6_* as the solvent, unless otherwise specified. The abbreviation dm in the ^1^H NMR spectra means doublet of multiplet. It describes more complex couplings observed at some aromatic hydrogens. IR spectra were acquired on FT-IR-ATR REACT IR 1000 (ASI Applied Systems) with diamond probe and MTS detector. Mass spectra were performed on LC–MS (Agilent Technologies 1200 Series equipped with Mass spectrometer Agilent Technologies 6100 Quadrupole LC–MS) and GC–MS (Agilent Technologies 6890N gas chromatograph with a 5973 Network mass-selective detector (Agilent, Waldbronn, Germany)). The course of the reactions was followed by TLC analysis (Merck Silica gel 60 F_254_). UV lamp (254 nm) and iodine vapours were used for the visualization of TLC spots. Melting points were measured by using Kofler apparatus or Barnstead Electrothermal IA9200 and are uncorrected. 4-Methoxybenzene-1-sulfonyl chloride (**1**) was purchased from Sigma-Aldrich.

**4-Methoxybenzene-1-sulfonyl chloride** (**1**), CAS [98-68-0], is a commercially available compound. This substance can be prepared in 88% yield from readily available anisole, sulfuryl chloride, H_2_SO_4_ and DMF according to the literature [10]. Compound **1** can be alternatively obtained from anisole in 81% yield by ClSO_3_H at 5 °C [11] or in 66% yield by ClSO_3_H in CHCl_3_ [12].

**Sodium 4-methoxybenzenesulfinate** (**2**), CAS [6462-50-6], is not a commercially available compound. Sulfinate **2** can be prepared from arylsulfonyl chloride **1** by treatment with Na_2_SO_3_, NaHCO_3_ at 65 °C in 98% yield [13] or in 67% yield [14]. Compound **2** has not been described. The physicochemical properties of compound **2** were not found in the SciFinder database. The Reaxys database contains its ^1^H NMR (300 MHz, CD_3_OD) [15] and IR spectra [16].

**1-(Ethylsulfonyl)-4-methoxybenzene** (**3**), CAS [7205-79-0], is not a commercially available compound from current suppliers. The Reaxys database contains different mp values for arylethylsulfone **3**: 40–41°C [EtOH] [16], 55–56 °C [EtOH/H_2_O] [17] (also in SciFinder), 57 °C [18] and 59 °C [19] together with three ^1^H NMR spectra, among them one with assigned solvent (CDCl_3_) [20]. No direct synthesis of **3** has been found in the SciFinder database. The Reaxys database describes five preparations for compound **3** from different but not very common substrates. Only one of them starting from 1,4-bis(ethylsulfonyl)benzene was published, with 65% yield [21]. None of the above syntheses were performed from sodium arylsulfinate **2** as we describe here.

**4-(Ethylsulfonyl)-1-methoxy-2-nitrobenzene** (**4**), CAS [51572-44-2], is not commercially available from Sigma-Aldrich, Merck or Acros Organics. No direct synthesis or experimental physicochemical properties have been described for this compound in the SciFinder database. In the Reaxys database only one mp, i.e., 119–120 °C was present [19] and no spectral data were available for **4**. One half reaction describing nitration of ethylsulfonylanisol with HNO_3_ was mentioned in the patent literature [19]. No yield was given in this record.

**5-(Ethylsulfonyl)-2-methoxy-1,3-dinitrobenzene** (**4a**) is an unknown compound. No physicochemical properties or spectral data were found either in the Reaxys or in SciFinder databases.

**5-(Ethylsulfonyl)-2-methoxyaniline** (**5**), CAS [5339-62-8], is a compound with only limited commercial availability. For this compound no synthesis can be found in the Reaxys and SciFinder databases. There were 37 reactions in Reaxys and 191 reactions in SciFinder described. In all cases compound **5** was exclusively a starting material or reactant. Experimental IR and ^1^H NMR (CDCl_3_) spectra for compound **5** were mentioned in the SciFinder database and originated from Bio-Rad Laboratories data collection [9].

**Synthesis of sodium 4-methoxybenzenesulfinate (2).** A stirred mixture of H_2_O/THF (200 mL, 10:1, v/v) was used to dissolve 18.30 g (145.2 mmol, 2.00 equiv) Na_2_SO_3_ and 12.21 g (145.3 mmol, 2.00 equiv) NaHCO_3_. Then the solution was cooled in an ice bath to 0 °C. At this temperature 15.00 g (72.6 mmol, 1.00 equiv) **1** was added portionwise to the mixture over 10 min. The reaction was stirred overnight, and the temperature was allowed to increase to rt. The reaction mixture was purified by extraction with CHCl_3_ (3 × 50 mL). Water from the separated aqueous layer was distilled off until a white solid material was formed. The solid mixture was triturated by MeOH (3 × 50 mL) and the remaining solid material containing the required salt **2** was dried by RVE, HV and yielded 14.10 g (72.6 mmol, 99.9%) of **2**. White solid; mp neither melting nor decomposition was observed until 300 °C [MeOH]; IR (neat) ν/cm^−1^: 3358 (m, br), 2959 (m), 2837 (m), 1591 (s), 1575 (m), 1490 (s), 1459 (m), 1441 (m), 1401 (w), 1299 (m), 1244 (s), 1188 (m), 1171 (m), 1082 (m), 1046 (m), 1029 (s), 974 (s), 835 (m), 824 (w), 815 (m), 793 (m), 722 (w); ^1^H NMR (300 MHz, DMSO*-d*_6_) δ 7.42 (dm, *J*(2,3) = 8.7 Hz, 2H, H-C(2)), 6.87 (dm, *J*(2,3) = 8.7 Hz, 2H, H-C(3)), 3.74 (s, 3H, MeO-); ^13^C NMR (75 MHZ, DMSO*-d*_6_) δ 159.0 (C4-O), 127.0 (C1-S), 125.5 (C2), 112.8 (C3), 55.0 (MeO-); ESIMS *m*/*z*: 171.1 [M − Na]^−^; Anal. calcd for C_7_H_7_NaO_3_S (194.18): C, 43.30; H, 3.63; S, 16.51; found: C, 43.15; H, 3.48; S, 16.22%.


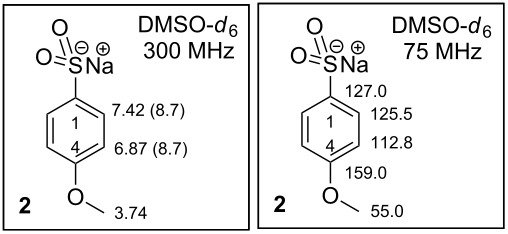


**Synthesis of 1-(ethylsulfonyl)-4-methoxybenzene (3).** A crude product from the previous reaction 12.20 g (62.8 mmol, 1.00 equiv) **2** was dissolved in 150 mL of MeOH. The solution was stirred and heated under reflux in an Ar atmosphere. When the reaction mixture started to boil, 12.6 mL (24.50 g, 157.1 mmol, 2.50 equiv) EtI was added dropwise over 10 min via syringe and septum. The reaction mixture was heated under reflux and monitored by TLC. After 2 h, the mixture was left to cool to rt and subsequently evaporated by RVE to give a pale yellow solid material. The crude product **3** was triturated with CHCl_3_ (3 × 30 mL), the suspension filtered and the organic solution concentrated by RVE to 1/10 of its original volume. The solution crystallized by standing in a refrigerator overnight to give white crystals. The crystals were filtered off and washed with cold hexane to yield 11.30 g (56.4 mmol, 89.8%) of the product **3**. Pale yellow solid; mp 54.9–56.4 °C [CHCl_3_] (lit. 55–56 °C [EtOH/H_2_O] [17]); IR (neat) ν/cm^−1^: 2972 (m), 2934 (m), 2848 (m), 1594 (m), 1578 (m), 1496 (m), 1473 (w), 1459 (m), 1417 (w), 1385 (w), 1317 (m), 1296 (m), 1263 (s), 1188 (w), 1137 (s), 1113 (m), 1088 (s), 1065 (w), 1050 (w), 1022 (m), 829 (m), 806 (m), 783 (m), 737 (m), 707 (m); ^1^H NMR (300 MHz, CDCl_3_) δ 7.83 (dm, *J*(2,3) = 9.0 Hz, 2H, H-C(2)), 7.03 (dm, *J*(2,3) = 9.0 Hz, 2H, H-C(2)), 3.89 (s, 3H, MeO), 3.09 (q, *J*(CH_2_,CH_3_) = 7.4 Hz, 2H, SO_2_CH_2_), 1.27 (t, *J*(CH_2_,CH_3_) = 7.4 Hz, 3H, SO_2_CH_2_C*H*_3_); ^13^C NMR (75 MHz, CDCl_3_) δ 163.7 (C4-O), 130.4 (C1-S), 130.0 (C2), 114.4 (C3), 55.7 (MeO), 50.8 (SO_2_CH_2_), 7.6 (SO_2_CH_2_*C*H_3_); ESIMS *m*/*z*: 201.1 [M + H]^+^; Anal. calcd for C_9_H_12_O_3_S (200.25): C, 53.98; H, 6.04; S, 16.01; found: C, 53.88; H, 5.90; S, 16.42%.


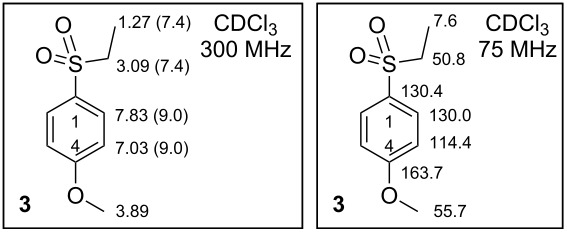


**Synthesis of 4-(ethylsulfonyl)-1-methoxy-2-nitrobenzene (4).** 1-(Ethylsulfonyl)-4-methoxybenzene (**3**) 11.30 g (56.4 mmol, 1.00 equiv) was dissolved in 140 mL of HNO_3_ (conc. 65%, w/w). The reaction mixture was heated to 100 °C and monitored by TLC. A complete conversion of starting material **4** was observed within 2 h. Then the mixture was left to cool to rt and carefully poured into 250 mL of ice-cold water. A white solid material precipitated. Then the suspension was washed with CHCl_3_ (3 × 60 mL). The combined organic phase was dried over Na_2_SO_4_ and filtered, and the solution was evaporated by RVE to yield 10.14 g (41.3 mmol, 73.3%) **4** in the form of a pale yellow solid material. Yellow solid; mp 118.5–119.7 °C [CHCl_3_] (lit. 119–120 °C [19]); IR (neat) ν/cm^−1^: 3076 (w), 2921 (m), 1606 (s), 1570 (w), 1532 (s, NO_2_), 1488 (w), 1475 (m), 1461 (m), 1350 (m), 1299 (s), 1279 (s), 1258 (m), 1236 (m), 1191 (w), 1161 (m), 1145 (s), 1109 (m), 1079 (m), 1051 (w), 1003 (m), 905 (w), 891 (m), 823 (m), 806 (m), 778 (m), 738 (m), 702 (m), 686 (m); ^1^H NMR (300 MHz, CDCl_3_) δ 8.37 (d, *J*(3,5) = 2.3 Hz, 1H, H-C(3)), 8.07 (dd, *J*(5,6) = 8.9, *J*(3,5) = 2.3 Hz, 1H, H-C(5)), 7.27 (d, *J*(5,6) = 8.9 Hz, 1H, H-C(6)), 4.07 (s, 3H, MeO), 3.15 (q, *J*(CH_2_,CH_3_) = 7.5 Hz, 2H, SO_2_CH_2_), 1.32 (t, *J*(CH_2_,CH_3_) = 7.5 Hz, 3H, SO_2_CH_2_C*H*_3_); ^13^C NMR (75 MHz, CDCl_3_) δ 156.6 (C1-O), 134.0 (C5), 130.5 (C2), 126.3 (C3), 114.1 (C6), 57.2 (MeO), 50.8 (SO_2_*C*H_2_), 7.5 (SO_2_CH_2_*C*H_3_), a signal for C4 is overlapped with one occurring in the range of 134.0–126.3 and, therefore, cannot be exactly assigned; GC–MS *m*/*z*: 245.1 (m, M^+^), 216.0 (s, M^+^ − Et), 214.0 (m, M^+^ − OMe), 200.0 (m, M^+^ − NO_2_ + H), 198.0 (m, M^+^ − NO_2_ − H), 168.1 (m, M^+^ − NO_2_ − OMe), 124.0 (m), 122.0 (m), 106.1 (m), 76.1 (m); Anal. calcd for C_9_H_11_NO_5_S (245.25): C, 44.08; H, 4.52; N, 5.71; S, 13.07; found: C, 43.97; H, 4.78; N, 5.45; S, 13.46%.


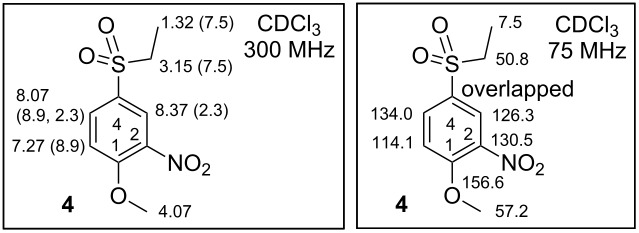


**Synthesis of 5-(ethylsulfonyl)-2-methoxy-1,3-dinitrobenzene (4a).** 1-(Ethylsulfonyl)-4-methoxybenzene 14.92 g (74.5 mmol, 1.00 equiv) **3** was portionwise dissolved in an ice-cold mixture of 120 mL H_2_SO_4_ (conc. 96%, w/w) and 120 mL of HNO_3_ (conc. 65%, w/w) at 0 °C. Afterwards the mixture was left to warm to rt (ca 2 h). After the above steps, TLC showed us only the presence of the starting material **3**. Therefore the reaction mixture was heated to 60 °C over 1 h. After this time TLC showed a new product. The reaction mixture was cooled to rt and poured into 500 mL of ice-cold water. A yellow solid material precipitated. The obtained suspension was washed with CHCl_3_ (3 × 60 mL). The combined organic phase was dried over Na_2_SO_4_, filtered and evaporation by RVE to yield 11.75 g (40.5 mmol, 54.4%) of **4a** in the form of a yellow powder. Yellow solid; mp 123.3–124.5 °C [CHCl_3_]; IR (neat) ν/cm^−1^: 3075 (m), 2974 (w), 1611 (m), 1536 (s, NO_2_), 1481 (m), 1457 (w), 1428 (w), 1406 (w), 1344 (m), 1314 (s), 1279 (m), 1260 (m), 1235 (w), 1211 (m), 1184 (m), 1130 (s), 1094 (m), 1047 (m), 968 (m), 927 (w), 912 (m), 890 (m), 785 (w), 772 (m), 737 (m), 711 (m), 686 (w); ^1^H NMR (300 MHz, CDCl_3_) δ 8.52 (s, 2H, H-C(4 and 6)), 4.15 (s, 3H, MeO), 3.23 (q, *J*(CH_2_,CH_3_) = 7.4 Hz, 2H, SO_2_CH_2_), 1.38 (t, *J*(CH_2_,CH_3_) = 7.4 Hz, 3H, SO_2_CH_2_C*H*_3_); ^13^C NMR (75 MHz, CDCl_3_) δ 151.5 (C2-O), 145.2 (C3), 134.7 (C5), 129.0 (C4), 65.3 (MeO), 50.8 (SO_2_*C*H_2_), 7.5 (SO_2_CH_2_*C*H_3_); ESIMS *m*/*z*: 290.1 [M]^−^; Anal. calcd for C_9_H_10_N_2_O_7_S (290.25): C, 37.24; H, 3.47; N, 9.65; S, 11.05; found: C, 36.89; H, 3.50; N, 9.15; S, 10.97%.


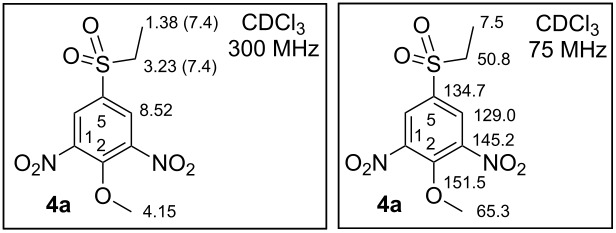


**Synthesis of 5-(ethylsulfonyl)-2-methoxyaniline (5)**. 4-(Ethylsulfonyl)-1-methoxy-2-nitrobenzene (**4**) 6.38 g (26.0 mmol, 1.00 equiv) was dissolved in 150 mL of EtOH. A catalytic amount of 10% Pd/C (64 mg, 1.00 w/w %) was added to the solution. The mixture was stirred under an atmosphere of H_2_ at 34 °C and regularly analysed by TLC. After 2 days the reaction was completed. The mixture was filtered through a small column filled with silica gel and the solution evaporated by RVE. The crude compound was dried by HV. Afterwards 5.01 g (23.3 mmol, 89.6%) of a solid powder was obtained as the pure product **5** according to the ^1^H NMR spectrum. For mp determination a crystallization of **5** from MeOH was performed. Solid; mp 102.9–103.8 °C [MeOH]; IR (neat) ν/cm^−1^: 3459 (s, NH_2_), 3366 (s, NH_2_), 3080 (w), 2966 (m), 2944 (m), 2924 (m), 2843 (m), 1616 (m), 1578 (m), 1504 (m), 1470 (m), 1458 (m), 1426 (m), 1375 (w), 1354 (w), 1303 (m), 1287 (s), 1277 (s), 1228 (s), 1190 (w), 1137 (s), 1108 (s), 1074 (m), 1046 (m), 1018 (s), 919 (m), 874 (m), 819 (m), 759 (m), 735 (m), 719 (s), 660 (m); ^1^H NMR (300 MHz, CDCl_3_) δ 7.25 (dd, *J*(3,4) = 8.4, *J*(4,6) = 2.3 Hz, 1H, H-C(4)), 7.19 (d, *J*(4,6) = 2.3 Hz, 1H, H-C(6)), 6.87 (d, *J*(3,4) = 8.4 Hz, 1H, H-C(3)), 4.15 (br s, 2H, NH_2_), 3.92 (s, 3H, MeO), 3.07 (q, *J*(SO_2_CH_2_,CH_3_) = 7.4 Hz, 2H, SO_2_CH_2_), 1.24 (t, *J*(SO_2_CH_2_,CH_3_) = 7.4 Hz, 3H, SO_2_CH_2_C*H*_3_); ^13^C NMR (75 MHz, CDCl_3_) δ 151.1 (C2-O), 137.2 (C1), 130.3 (C5), 119.3 (C4), 113.3 (C6), 109.9 (C3), 56.0 (MeO), 50.9 (SO_2_*C*H_2_), 7.8 (SO_2_CH_2_*C*H_3_); ESIMS *m*/*z*: 216 [M + H]^+^; Anal. calcd for C_9_H_13_NO_3_S (215.27): C, 50.21; H, 6.09; N, 6.51; S, 14.90; found: C, 50.50; H, 6.15; N, 6.75; S, 14.77%.